# Analysing Humanly Generated Random Number Sequences: A Pattern-Based Approach

**DOI:** 10.1371/journal.pone.0041531

**Published:** 2012-07-23

**Authors:** Marc-André Schulz, Barbara Schmalbach, Peter Brugger, Karsten Witt

**Affiliations:** 1 Department of Psychiatry and Psychotherapy, RWTH Aachen University, Aachen, Germany; 2 Department of Neurology, Christian Albrecht University, Kiel, Germany; 3 Neuropsychology Unit, Department of Neurology, University Hospital Zurich, Zurich, Switzerland; University of Swansea, United Kingdom

## Abstract

In a random number generation task, participants are asked to generate a random sequence of numbers, most typically the digits 1 to 9. Such number sequences are not mathematically random, and both extent and type of bias allow one to characterize the brain's “internal random number generator”. We assume that certain patterns and their variations will frequently occur in humanly generated random number sequences. Thus, we introduce a pattern-based analysis of random number sequences. Twenty healthy subjects randomly generated two sequences of 300 numbers each. Sequences were analysed to identify the patterns of numbers predominantly used by the subjects and to calculate the frequency of a specific pattern and its variations within the number sequence. This pattern analysis is based on the Damerau-Levenshtein distance, which counts the number of edit operations that are needed to convert one string into another. We built a model that predicts not only the next item in a humanly generated random number sequence based on the item′s immediate history, but also the deployment of patterns in another sequence generated by the same subject. When a history of seven items was computed, the mean correct prediction rate rose up to 27% (with an individual maximum of 46%, chance performance of 11%). Furthermore, we assumed that when predicting one subject′s sequence, predictions based on statistical information from the same subject should yield a higher success rate than predictions based on statistical information from a different subject. When provided with two sequences from the same subject and one from a different subject, an algorithm identifies the foreign sequence in up to 88% of the cases. In conclusion, the pattern-based analysis using the Levenshtein-Damarau distance is both able to predict humanly generated random number sequences and to identify person-specific information within a humanly generated random number sequence.

## Introduction

In a Random Generation Task (RGT) the subject is asked to generate a random sequence of items. The most popular variant is the Random Number Generation Task (RNGT) in which subjects are requested to repeatedly pick a number from a given range (typically the digits 1 to 6 or 1 to 9). Earlier research showed that human beings are far from perfect in producing random sequences. They typically avoid number repetitions and systematically deviate from mathematical randomness [1]. The RNGT has become a famous tool for research focusing on working memory, more specifically on the role of the central executive component of working memory and also on the supervisory attentional system in healthy subjects [2], [3], [4], [5]. The task has been applied to healthy subjects and patients suffering from frontal lobe damage [6], Alzheimer disease [7], Parkinson’s disease [6], [8], schizophrenia [9], [10], [11] or other diseases affecting the central nervous system [9], [10], [11], [12]. These studies showed impairments on different measures of randomness in healthy subjects but more profound impairments in patients.

The RNGT is a demanding task as it requires the subject to utilise a variety of criteria that constitute perceived randomness. There are established methods to analyse humanly generated random number sequences (for a review see [14]) and most of them describe their deviation from mathematical randomness. Methods representative for the currently taken approach are the standard information theory approach using entropy and redundancy, the Evans Random Number Generation Index which describes the distribution of pairs, the coupon score which is the mean number of responses recorded before all response alternatives occurred and counting scores which score habitual counting tendencies [13]. Most of these measures are inter-correlated and a factor analysis of a subset of measures identified three main dimensions: cycling, seriation and repetition [14].

**Figure 1:**
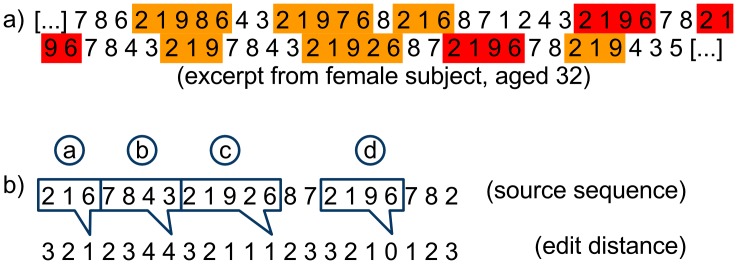
A demonstration of the pattern based approach. (A) In this sequence, the pattern (2, 1, 9, 6), marked in red, is predominant. Variations of this pattern are marked in orange. (B) demonstrates the concept of the edit distance according to Damerau-Levenshtein. The edit distance indicates the number of edit operations necessary to convert the humanly generated random number sequence at any position into a given pattern. A distance of 0 marks a perfect match (d). At a distance of 1, one edit operation is needed to convert the sequence string into the pattern (a: deletion, c: insertion). If the patterns do not match to the given string of the sequence, up to 4 edit operations are needed. Therefore the score is 4 (b). The inverse numbers of the edit operations are added up and this score represents the mathematical “affinity” of a given pattern to the humanly generated random sequence with a lower score for patterns with diminished “affinity” to the original sequence.

We introduce a novel approach to analyse humanly generated random number sequences without referring to mathematical randomness. We based our analysis on two principles. First, subjects are aware of the last choices they made. As the subsequent choices are dependent on their last choice, a history of choices must be stored in the brain, which has a limited memory capacity. Second, subjects utilise a certain set of transition rules which determine, with regard to item history and the set of alternatives, how to proceed with the number sequence. The generated sequences are not mathematically random, but the brain obviously follows rules. If these rules are fixed and switches between rules are limited, the mathematical concept of a higher order Markov chain gives us a formal description of the underlying processes. A Markov chain describes a system that switches between a fixed number of states, here from one digit to another, in a chainlike manner. Higher order Markov chains tend to produce repetitive patterns. Since these patterns reveal information about the original Markov chain, we use them as the starting point of our analysis. Figure 1a shows an example of phrasal structures (patterns) in humanly generated random number sequences. The predominant pattern (e.g. 2, 1, 9, 6) is well-preserved. However, it makes some modifications (e.g. inserted numbers) and the sequence also breaks off into other patterns. To explore these patterns of numbers, we use an approximate string-matching technique to find approximate matches to a pattern in a string. We use the Damerau-Levenshtein distance as a mathematical approach that counts the “distance” between two strings, in our case a sequence of numbers counting the minimum number of operations needed to transform one string into the other (figure 1b) [15]. An edit operation is defined as an insertion, deletion, or substitution of a single character (number), or a transposition of two adjacent characters of a string (pattern). As these edit operations can be considered elementary, this method is used here to search for patterns in humanly generated random number sequences.

If the utilized Markov chain (order, transition probabilities, and thus, also patterns) has been established in a person’s choice of numbers, two predictions can be made: First, a detailed analysis of the individual patterns should make it possible to predict the future course of the generated number sequence of that person. Secondly, if the patterns show inter-individual differences, and to a lesser degree, intra-individual differences, the pattern analysis may allow us to identify a person on the basis of his or her patterns. The aim of the present study is to build a pattern analysis that predicts an individual’s humanly generated random number sequences and even makes it possible to identify a person using complex information provided by his or her individual patterns.

## Methods

### Subjects and Procedure

Participants were students (n = 20, 18 female, aged 20–32 years, mean 25.8 years, SD +/−2.5 years) recruited from the Christian Albrecht University Kiel, Germany. All subjects were non-smokers and reported that they were in good health, without any systemic diseases, and without any history of neurologic or psychiatric disease. They had not taken any medication or consumed any illicit drugs in a 3-month interval before the study. The study procedure was approved by the local ethics committee of the Christian Albrecht University and subjects gave written informed consent before participating in the study.

For the RNGT, subjects were instructed to generate two series of 300 single digits between 1 and 9 in random order. This was paced by a tone (1 Hz). Participants rested for 7 minutes after the first series of the RNGT. The concept of randomness was explained using standard procedures (using instructions based on an analogy of selecting and replacing numbered table tennis balls from a shoe carton). A test trial was undertaken.

### Pattern Analysis

We defined a RNGT of length *l* as the generation of a sequence of *l* drawn from a finite set (target quantity *D*) with replacement. The generated random sequence we called *(z_i_), z_i_* ∈ *D*. As a *pattern m* of length *n* we denoted any n-tuple *(m_i_),m_i_* ∈ *D*. In our example (figure 1), a pattern with the length of *n* = 4 (2, 1, 9, 6) is shown. To mathematically measure “the similarity” of a pattern to a given sequence we introduced the function *s*(*m, z*), which takes a sequence and a pattern as input and returns a real number. The value *s*(*m, z*) indicates how often the pattern is contained in the sequence. We call it score *s* of pattern *m* on random sequence *z*. To calculate the score of a pattern on every position of the humanly generated random number sequence, the Levenshtein-Damerau distance d of the pattern to the corresponding part of the random sequence was computed.
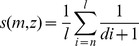



The sum of the inverses of the distances, divided by the sequence length, results in the *score* of the pattern on the random sequence. Figure 1b illustrates this procedure.

### Prediction

In this first application of the pattern analysis approach, we tested whether information on predominant patterns of a subject would be sufficient to predict future sequences generated by this subject. In our attempt to predict a random sequence we applied the following procedure: For each subject we used one random sequence (*x_i_)* consisting of 300 numbers to generate a statistical model which was applied to predict the second sequence *(y_i_)*. The statistical model is essentially a code book containing the scores for each pattern of a given length *n*. We computed *s(m,x)* for all patterns *m* of length *n.* Thus, when we tried to predict the next element of *y,(y_k_*) we chose the element which, combined with the past *n-1* elements of *(y_k_*), resulted in the pattern most often used in sequence *(x_i_*). We defined *q(j) = s((y_k-(n-1)_*,…,*y_k-1_,j),x)* so that we could call the element *j* ∈ *D* which maximized *q(j),* the best estimate for the next item of the sequence. Of course this approach is not limited to one source sequence and *q(j)* can be computed from any number of given sequences. The following example should illustrate this method: Let the sequences consist of numbers picked from *D = {1,…,9}*. Let the source sequence be *q(x_i_) = (1,2,3,4,1,3,2,1,2,3,6,4,9,1,2,3)*. This sequence is created around the pattern *(1,2,3)*. We analyse the source sequence for patterns of length *n = 3* (that is a computed history h of 2 items). Let the target sequence be *(y_i_) = (6,4,1,2)*. We wish to predict the next element. Thus we calculate the scores (*x*) for the patterns *(1,2,i)* for every *i* in *{1,…,9}* and choose the number *s* with the minimal score as the prediction for the successor of *y_4_*.

A history of zero equals a majority classifier (always guessing the most frequently occurring item). For each subject we use the first sequence *z_a_* to predict the next number in a sequence at every point of the second sequence *z_b_* and vice versa. We calculate the rate of exact hits ζ*(z_a_,z_b,_h)* for a computed history of 0,.,10 items with 10 being an arbitrary upper bound.

**Figure 2:**
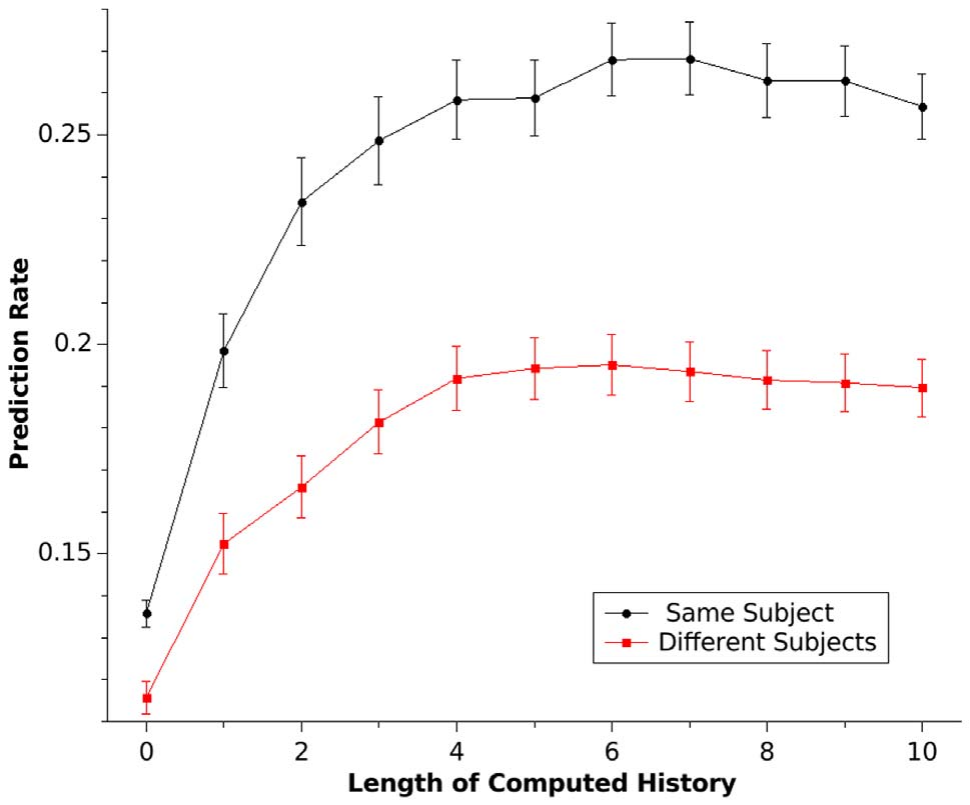
The prediction rate is shown as a function of the length of computed history (*h*). The prediction rate for sequences from the same subject is shown in black; the predication rate for sequences of different subjects is shown in red. The predication rate increases with the length of the computed sequence history. As *h* increases, the next sequence element can be predicted more precisely and with a history of length *h* = 7, 27% correct predictions of same-subject sequences can be made (chance performance  = 11%). Error bars represent SEM.

### Identification

In a second application of the pattern analysis approach, we tested the hypothesis that patterns derived from a humanly generated random number sequence show inter-individual differences. This hypothesis points to the fact that the Markov transition matrices (and/or the order of the Markov chain) of different subjects would not be identical. Both random number sequences of the 20 subjects were analysed. We calculated ζ*(z_a_,z_b,_h)* for every possible “sequence-sequence” combination for all sequences in our data set and compared ζ for sequences originating from the same subject (within-subject analysis) and ζ for sequences originating from different subjects (between-subject analysis). Additionally, we assumed that, when predicting one subject’s sequence, predictions based on statistical information from the same subject should yield a higher success rate than predictions based on statistical information from a different subject. Thus we took three sequences, one reference sequence, one sequence of the same subject and one of a different subject, and tried to predict the reference sequence. If it was ζ*_same_ >* ζ*_different_*, we considered the origin of the sequence to be identified correctly. We checked ζ*_same_ >* ζ*_different_* for every sequence triplet in the data set and called the relative frequency of correct identifications *η(h)* for any given *h*. As in the prediction experiment, we analysed the history lengths *h* from 0 to 10.

**Figure 3:**
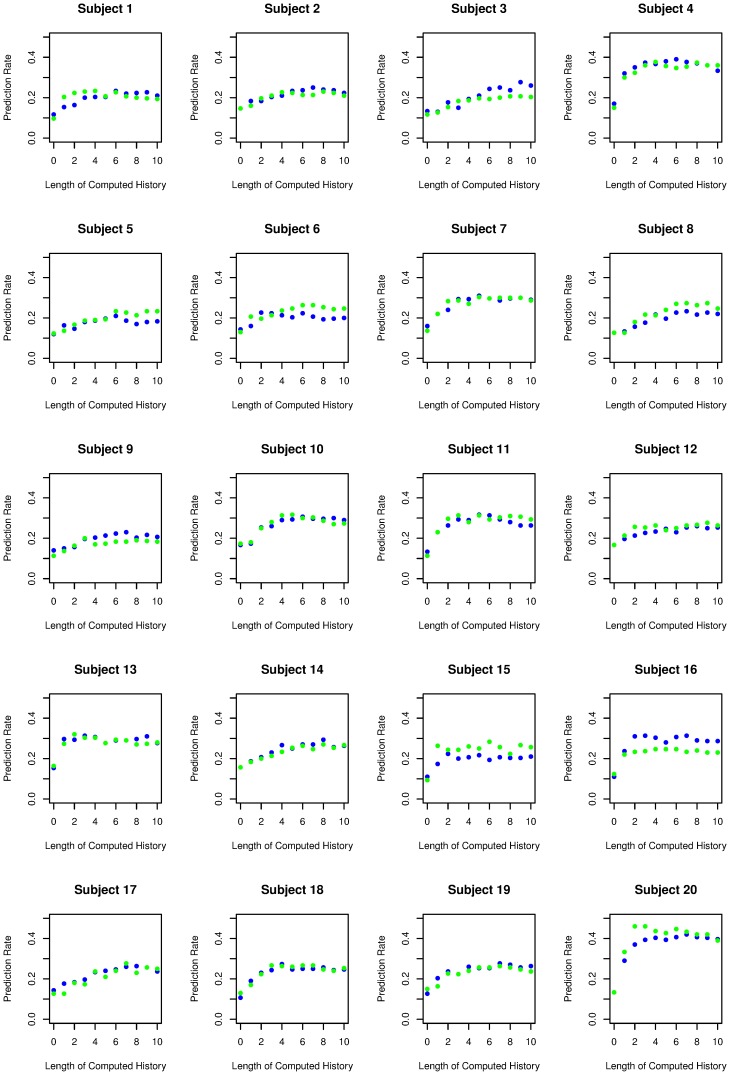
The success in predicting the second sequence based on the first sequence (green) and for the first sequence based on the second sequence (blue) is displayed for every subject. In some subjects (e.g. subjects 2, 4, 10, 14 and 18) prediction rates of both sequences are almost the same, whereas prediction rates differ in other subjects (e.g. subjects 16 and 20). The differences shown in subjects 16 and 20 might indicate that the subjects changed their strategies while generating random number sequences.

**Figure 4:**
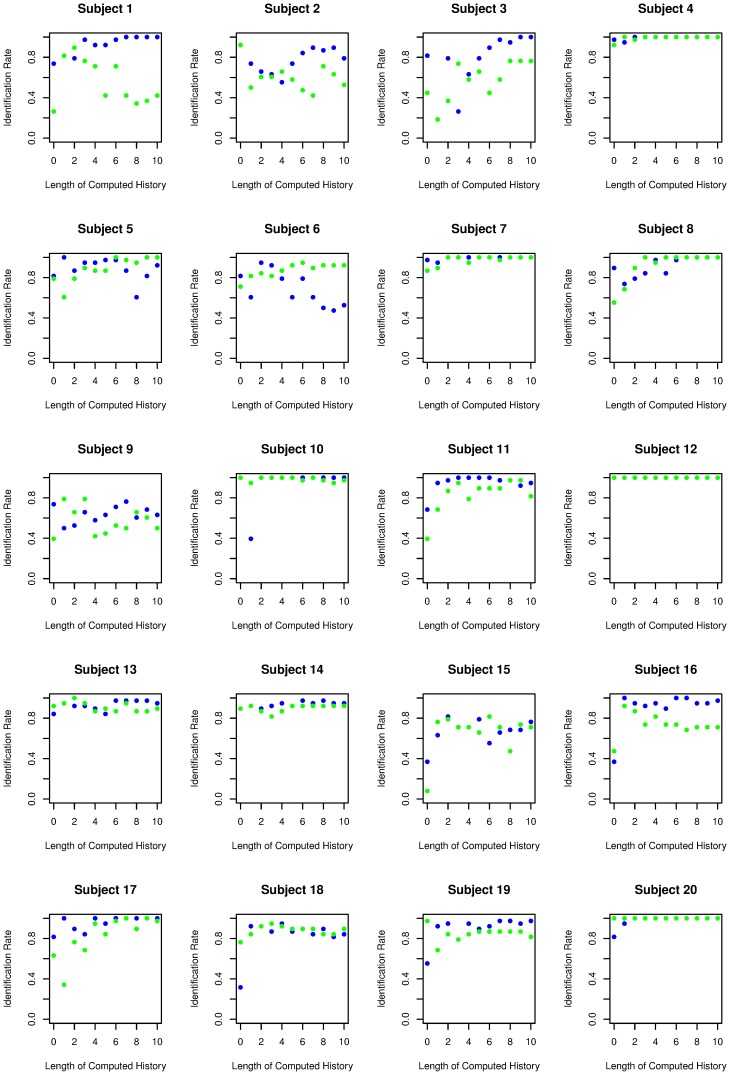
Identification rates to identify the second sequence based on the pattern analysis of the first sequence (green) and for the first sequence based on the pattern analysis of the second sequence (blue) are displayed for every subject (within-subject design). Identification rates for the two same-subject sequences (e.g. 7, 12 and 20) are very high and show great similarity, whereas identification rates differ when contrasting sequences with other subjects (e.g. 9 and 15).

**Figure 5:**
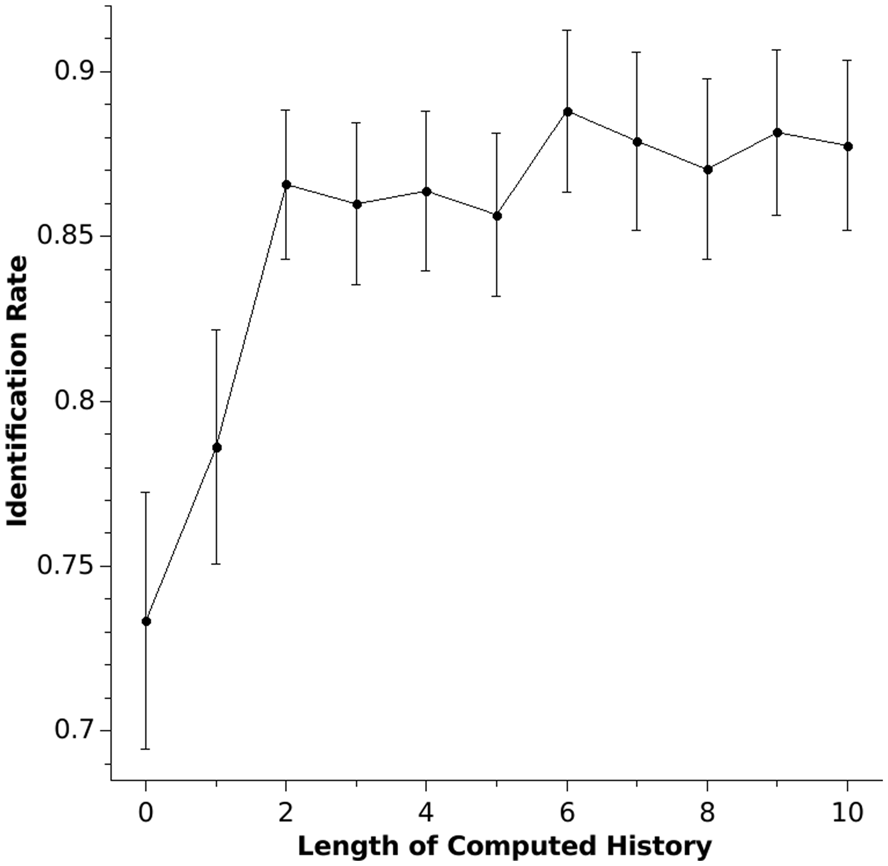
When provided with two sequences from the same subject and one from a different subject, a pattern analysis of the randomly generated sequences of 300 numbers identifies the alien sequence in up to 88% of the cases (*h* = 6). Error bars represent SEM.

## Results

### Prediction

The results of the prediction rate are shown in figure 2 (“same subject”). The mean prediction rate depends on the length of the computed history of the sequence and ranges between 14% correct predictions at *h* = 0 and a maximum of 27% of correct predictions at *h* = 7. The prediction rate with *h* = 0 is significantly higher than chance performance (T = 7.5, p<.001) and the difference between chance performance and the prediction rate increases with the length of computed sequence history (ANOVA with computed sequence length as a repeated variable, T = 15.9, p<.001). Figure 3 shows the prediction rates for individual subjects. Subjects differ in their overall predictability, ranging from 14% to 46% in trials with a computed history of the sequence *h*>2. In some subjects occasional differences in predictability between the two prediction directions are evident (see subjects 16 and 20, figure 3). These differences might be the result of a change in generation strategies.

### Identification

Figure 2 shows the prediction rate in relation to the length of the sequence history analysing two sequences from the same subjects (within-subject analysis) and analysing sequences between different subjects (between-subject analysis). Without any information about the sequence history (*h* = 0), the prediction rate of a sequence from the same subject is significantly higher than the prediction rate of a sequence of another subject (T = 5.1, p<.001), indicating that the subjects had strong preferences for individual digits. The differences between the prediction rate for the sequence of the same subject and the prediction rate for a different subject increase as the length of sequence history increases (statistical maximum at *h = *10, T = 10.3, p<.001). Both the prediction rates for the same subject and the prediction rates for different subjects increase with a greater length of number history (ANOVA with repeated measurements F = 34.2, p<.001). The increase in prediction rate in different subjects might reflect the fact that common rules such as repetition avoidance exist in most healthy subjects [8]. This ANOVA also shows a significant *h* × subject (prediction for the same subject vs. prediction for a different subject) interaction (F = 25.9, p<.001), demonstrating better prediction in sequences of the same subject with increasing length of number history. Individual identification rates are given in figure 4. The individual analysis showed nearly perfect identification in some patients (subject 4, 12, 20), whereas the identification rate is much lower in other subjects, although still above chance performance. The maximal correct identification rate (the ability to discriminate between the same and a different subject sequence; hit rate) is 88%, whereas chance performance is 50%. These results are shown in figure 5. They indicate that humans have strong individual preferences with respect to individual digit choices as well as digit patterns. n.

## Discussion

Both of our predictions were confirmed: Firstly, we showed that a detailed analysis of the individual’s patterns predicts the future course of the sequence for that person. The prediction rate reaches a maximum of 45% correct predictions for individual cases, far above the chance performance of 11% correct prediction in a number setting of 1 to 9. In the second hypothesis, we assumed that the patterns in the number sequence would differ less between same subject trials than trials between different subjects. When provided with two sequences of the same subject and one of a different subject, we tried to identify the alien sequence based on the intersequence predictability. The pattern-based approach showed a correct identification rate with a maximum of 88% correct identifications. The high identification rate is based on strong individual preferences and strategies in the generation of numbers. The analysis detects person-specific patterns - a biometric feature - like a mental fingerprint shining through in the humanly generated random number sequences. Both hypotheses are two sides of one coin and can be seen by using an algorithm that characterises specific patterns in a humanly generated random number sequence. This algorithm can not only predict the future course of a person’s number sequence, but also makes it possible to identify a person on the basis of his or her individual patterns. These results emphasize the idea of a complex hidden Markov rule system that underlies humanly generated random number sequences. Randomness can be described using Markov rule systems that connect all items of a given range (e.g. *D = {1,…,9}*) and every route within this Markov rule system has the same probability of being chosen. This model resembles Monte Carlo systems in computer sciences [16], [17]. Humans seem to choose individual routes within a Markov rule system when generating individual patterns. A set of universal patterns generated by rules that are not specific for one person such as repetition avoidance [8] explains the fact that information from one subject’s sequence can partly predict another subject’s sequence. Both prediction rate and correct hits in the identification experiment increased significantly with increased length of computed history, indicating that the pattern-based approach extracts additional non-trivial and person-specific information from the sequences that drive prediction and classification scores.

The pattern analysis based on the Damerau-Levenshtein distance simply counts the number of edit operations necessary to convert one pattern into another. Despite this simple principle, prediction rate and identification rate are remarkably high. Further modifications might improve this analysis. However, in the present study, we searched for preferential patterns, but did not explicitly analyse patterns that are neglected or avoided by a participant. The avoidance of specific patterns might also be person-specific. Our definition of the prediction rate only includes exact hits but may well be extended to consider probability estimates to give a more accurate assessment of the prediction quality. Furthermore, the number and the type of edit operation used to calculate the Levenshtein-Damerau distance might also include person-specific information. The weighting of edit operations might also improve prediction and identification rates. Additional information can be extracted from the humanly generated random number sequences that might enable us to find further mathematical correlates for cognitive functions and to develop computational models of the cognitive functions involved in random number generation.

There is further need to explore the place of pattern analysis in the field of random number generation research. Firstly, statistics correlating the results of a pattern analysis and the established indices of random number generation are definitely needed. Secondly, although we showed that the individual patterns are at least partially stable for a short time interval of seven minutes, it remains to be seen whether these patterns are stable over a longer time interval. Thirdly, due to the fact that subjects must keep their number choices in their working memory, further analysis of pattern length and working memory capacity might be interesting. And finally, pharmacological interventions, physiological stressors to the brain such as sleep deprivation [18], [19] and various types of brain damage might alter individual human patterns in different ways. This raises the question whether a pattern analysis might also be a useful tool for clinical neuropsychology, psychiatry and neurology.
